# Focal Takotsubo Syndrome Mimicking a Distal Coronary Pathology: A Case Report

**DOI:** 10.7759/cureus.109994

**Published:** 2026-05-31

**Authors:** Mohammed N Taha, Abdullah S Kadhim

**Affiliations:** 1 Internal Medicine, Baptist Health Medical Center-North Little Rock, North Little Rock, USA; 2 Internal Medicine, Al-Sha'ab General Hospital, Baghdad, IRQ

**Keywords:** acute coronary syndrome mimic, focal variant takotsubo syndrome, non-obstructive coronary arteries, stress-induced cardiomyopathy, takotsubo syndrome

## Abstract

Takotsubo syndrome (TTS) is a transient stress-induced cardiomyopathy. It typically occurs following emotional or physical stressors. Many variants are described, the most common being the apical ballooning; however, atypical variants such as focal TTS are rare and can mimic acute coronary syndromes. We report the case of a 49-year-old woman presenting with chest pain and marked troponin elevation who underwent coronary angiography with left ventriculography demonstrating non-obstructive coronary arteries, inferior apical hypokinesis, and a subtle distal left anterior descending artery abnormality. Serial troponin testing confirmed a peak high-sensitivity troponin of 1,967 ng/L. In the context of emotional stress, absence of obstructive coronary disease, and atypical angiographic features inconsistent with plaque rupture or spontaneous coronary artery dissection, a diagnosis of focal TTS was considered most consistent. This case highlights the diagnostic challenges in identifying focal variants of Takotsubo cardiomyopathy.

## Introduction

Takotsubo syndrome (TTS) is an acute cardiac syndrome characterized by transient left ventricular systolic dysfunction in the absence of obstructive coronary artery disease. This cardiomyopathy typically happens following emotional or physical stressors [1]. There are multiple types of Takotsubo cardiomyopathy. The most commonly observed pattern is apical ballooning. Other reported variant types include mid-ventricular, basal, focal, and the global hypokinesis type [2-5].

Among TTS cases, the focal variant is particularly uncommon, representing approximately 1.2-1.5% of all TTS diagnoses [1-5], and is often under-recognized because it can closely mimic acute myocardial infarction or other causes of myocardial infarction with non-obstructive coronary arteries (MINOCA) [5-7].

The most common signs and symptoms of TTS include chest pain, shortness of breath, and syncope, making it difficult to distinguish from acute myocardial infarction [3].

## Case presentation

**Figure 1:**
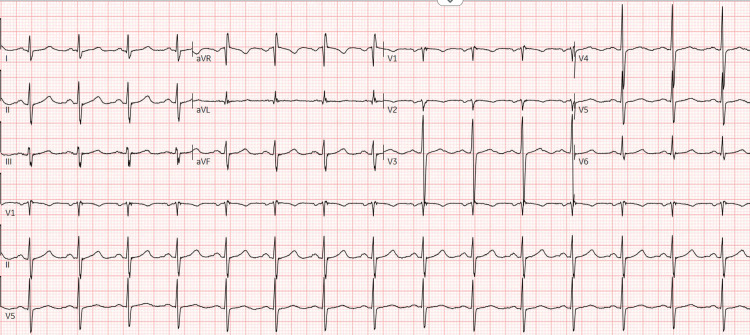
Electrocardiogram showing sinus rhythm with narrow QRS complexes, normal axis, and no acute ST-T-wave changes

A 49-year-old woman with a history of smoking presented to the emergency department with acute chest pain. She reported significant emotional stress in the weeks preceding the presentation. On arrival, vital signs were stable. Electrocardiography demonstrated a normal sinus rhythm without ischemic ST-segment deviation or T-wave abnormalities (Figure 1).

**Figure 2:**
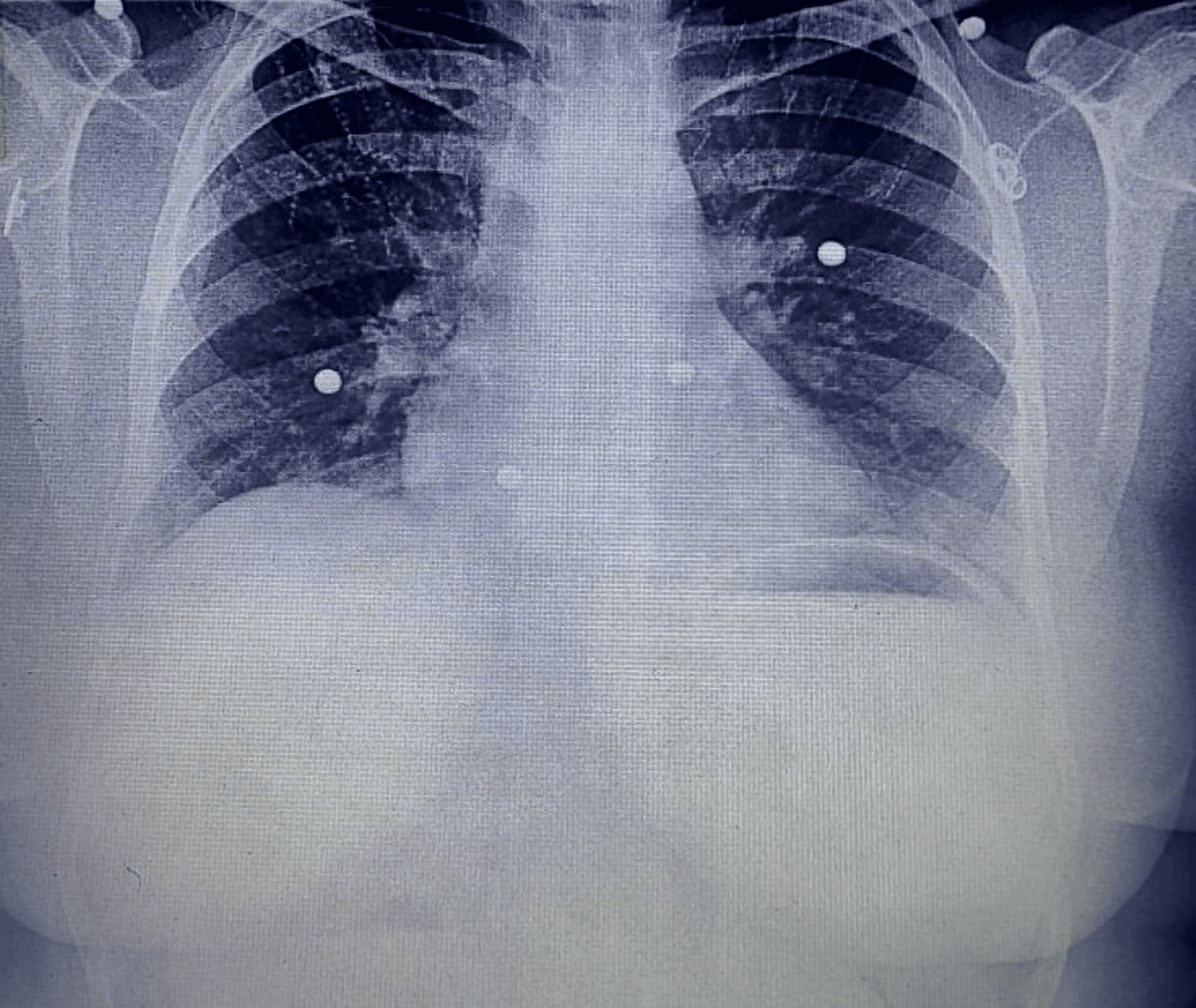
Posteroanterior view chest X-ray demonstrating no acute cardiopulmonary findings

Chest radiography was unremarkable (Figure 2).

Serial laboratory evaluation (Table 1) revealed rising high-sensitivity cardiac troponin levels on repeat testing, with an initial value of 1,169.1 ng/L and a peak of 1,967 ng/L on subsequent measurement, confirming significant myocardial injury.

**Table 1:** Laboratory investigations at presentation Notable findings include elevated high-sensitivity troponin, cholesterol, triglycerides, and LDL. WBC: white blood cell; BUN: blood urea nitrogen; CK: creatine kinase; LDL: low-density lipoprotein; TSH: thyroid-stimulating hormone

Test	Value	Reference range
Hematology		
WBC	5.8×10³/µL	4.5-11.0×10³/µL
Hemoglobin	14.9 g/dL	12-16 g/dL
Platelets	320×10³/µL	150-400×10³/µL
Metabolic/renal		
BUN	12 mg/dL	7-18 mg/dL
Sodium	138 mEq/L	136-145 mEq/L
Magnesium	2.19 mg/dL	1.560-2.520 mg/dL
Potassium	3.5 mEq/L	3.5-5.1 mEq/L
Creatinine	0.61 mg/dL	0.57-1.11 mg/dL
Cardiac		
High-sensitivity troponin (initial)	1,169.1 ng/L ▲ HIGH	<14.0 ng/L
High-sensitivity troponin (peak)	1,967 ng/L ▲ HIGH	<14.0 ng/L
CK, total	161 IU/L	29-168 IU/L
Lipids and metabolic		
Cholesterol	248 mg/dL ▲ HIGH	0-200 mg/dL
Triglycerides	244 mg/dL ▲ HIGH	0-150 mg/dL
LDL	173 mg/dL ▲ HIGH	0-99 mg/dL
A1C	5.4%	4.1-5.6%
Thyroid		
TSH	1.136 mIU/L	0.350-4.940 mIU/L

Given the ongoing chest pain and significant biomarker elevation, an invasive strategy was pursued, and the patient underwent urgent coronary angiography. She was started on aspirin, a beta-blocker, a statin, and heparin while awaiting the angiography results. Angiography revealed a large-caliber left anterior descending (LAD) artery giving rise to a medium-sized high first diagonal branch and continuing to the apex, where it bifurcated to supply the distal half of the inferior wall, consistent with wrap-around LAD anatomy. Mild proximal myocardial bridging was noted without evidence of hemodynamically significant compression. No obstructive coronary artery disease or plaque rupture was identified.

A subtle, ill-defined focal radiolucency was observed in the medial aspect of two distal apical LAD branches, best appreciated in the right anterior oblique caudal projection. Bracketing areas of increased contrast density suggested vessel tortuosity rather than a discrete stenotic lesion or intimal flap. Given the extremely distal location and focal nature of the finding, intracoronary imaging with optical coherence tomography (OCT) or intravascular ultrasound (IVUS) was considered; however, it was deemed technically unfeasible due to the extremely distal vessel location and the small caliber of the involved branch, which would have posed a prohibitive risk. Spontaneous coronary artery dissection (SCAD) or atherothrombotic infarction was therefore considered less likely based on angiographic appearance. In the context of antecedent emotional stress and non-obstructive coronaries, focal TTS was favored.

**Figure 3:**
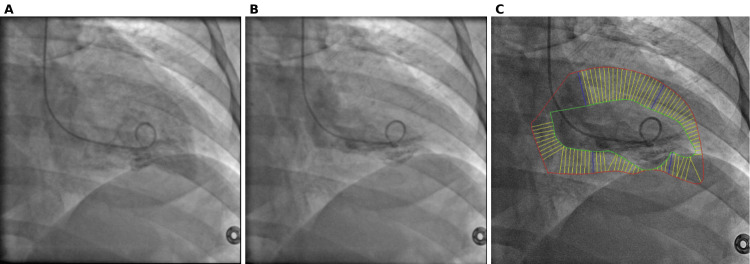
Left ventriculography demonstrating focal Takotsubo syndrome. (A) End-diastolic frame showing the left ventricular contour at maximum filling. (B) End-systolic frame demonstrating the reduced inward wall motion in the inferior apical segment, reflecting focal hypokinesis. (C) Quantitative wall motion analysis overlay. The red contour represents the end-diastolic outline, while the green contour represents the end-systolic outline, with yellow chords illustrating reduced shortening in the inferior apical region

Left ventriculography demonstrated inferior apical hypokinesis, consistent with a focal wall motion abnormality (Figure 3).

Following angiography, focal TTS was considered the most likely diagnosis. As there was no obstruction, no coronary intervention was done. The patient was treated with a beta-blocker and an angiotensin receptor blocker (ARB) as tolerated. She remained hemodynamically stable, improved clinically, and was discharged home with outpatient follow-up.

## Discussion

This case shows a significant diagnostic challenge both clinically and angiographically. The focal variant is rare and can closely mimic myocardial infarction. Registry data demonstrate that focal variants of TTS are increasingly recognized, likely reflecting improved awareness and diagnostic imaging [1,5,8].

Takotsubo cardiomyopathy can be diagnosed using the modified Mayo Clinic criteria, which require transient hypokinesia or akinesia, absence of obstructive coronary artery disease, new electrocardiogram (ECG) changes or troponin elevation, and absence of myocarditis or pheochromocytoma [9].

In this case, the ECG showed a normal sinus rhythm. There are no absolute ECG findings specific to diagnosing TTS. However, some changes can appear for multiple reasons. It has been reported that up to 21-49% of patients present with ST-segment elevation at presentation, usually in the precordial leads, and T-wave inversions are also frequently observed [3,10].

Although troponin elevation is typically modest in TTS, substantial elevations have been reported, particularly when compared with the degree of ventricular dysfunction, and should not exclude the diagnosis [1,11,12].

The inferior apical hypokinesis seen on ventriculography is a pattern consistent with focal TTS, as the focal variant characteristically involves a localized wall motion abnormality rather than the diffuse apical ballooning seen in classic TTS. The incidences of TTS types on left ventriculography during catheterization are apical in 81.7%, mid-ventricular in 14.6%, basal in 2.2%, or focal in 1.5% of patients [1,13].

An important diagnostic consideration in this case was SCAD, an important cause of acute coronary syndrome in women. SCAD findings are usually described in angiographic patterns, including long smooth narrowing, intramural hematoma, or visible intimal flaps, none of which were present on angiography [14-16]. Additionally, the focal and extremely distal nature of the angiographic abnormality, along with contrast "bracketing" suggestive of tortuosity, was atypical for SCAD. Intracoronary imaging with OCT or IVUS, which would have provided definitive characterization of the vessel wall, was not performed due to the extremely distal vessel location and technical constraints. Accordingly, while SCAD was considered less likely based on the overall clinical and angiographic picture, it cannot be completely excluded in the absence of intracoronary imaging or cardiac magnetic resonance imaging (CMR) [14,15].

CMR is increasingly emphasized in consensus documents as a critical tool in distinguishing TTS from myocardial infarction and myocarditis, typically demonstrating myocardial edema with absent ischemic-pattern late gadolinium enhancement [7,17,18]. Follow-up imaging demonstrates that recovery of ventricular function remains a cornerstone of TTS diagnosis and prognostication [3,4].

Regarding management, this patient was treated in accordance with guideline-directed care for suspected acute coronary syndrome at presentation, with aspirin, beta-blocker, statin, and anticoagulation. She was then maintained on a beta-blocker and ARB following the confirmation of non-obstructive disease to support left ventricular recovery. Regarding long-term management, some studies suggest that angiotensin-converting enzyme inhibitors (ACEIs)/ARBs can improve one-year survival, while others show no benefit for survival or recurrence [4]. However, other studies showed that beta‐blockers and ACEIs/ARBs do not significantly affect recurrence [19]. Therefore, because of pharmacological uncertainty, further large-scale prospective studies are required.

Recent registry analyses focused on focal TTS confirm that it is rare and demonstrate the importance of careful imaging assessment and exclusion of alternative diagnoses [6,7], which is crucial to avoid misdiagnosis and unnecessary coronary interventions.

This case has several limitations. CMR was not performed, which would have provided stronger confirmation of TTS by demonstrating myocardial edema without ischemic-pattern late gadolinium enhancement. The extremely distal location of the angiographic abnormality prevented the use of intracoronary imaging (OCT or IVUS). Since distal SCAD can occasionally present without typical angiographic findings, its diagnosis cannot be definitively excluded in this case. Additionally, formal follow-up echocardiography confirming the recovery of wall motion abnormalities, a cornerstone of TTS diagnosis, was not documented in this report. These limitations leave open the possibility of alternative diagnoses, though the overall clinical picture remains most consistent with the focal variant of TTS.

## Conclusions

Focal TTS is a rare TTS variant but remains an important mimic of acute coronary syndrome. This case demonstrated the importance of recognizing atypical TTS presentations during angiography assessment, particularly in patients with emotional stressors, non-obstructive coronary angiography, together with wall hypo- or akinesia, and angiographic findings inconsistent with classic plaque rupture or SCAD. Distal SCAD should remain a consideration when angiographic findings are inconclusive and definitive exclusion by intracoronary imaging or CMR is not possible. Although the overall clinical and imaging profile favored focal TTS, the absence of confirmatory CMR limits definitive diagnostic certainty. Increased awareness of focal TTS and careful multimodality assessment are important for accurate diagnosis and appropriate management of patients presenting with myocardial infarction-like syndromes and non-obstructive coronary arteries. Management of TTS remains challenging, and a large cohort study is required to further clarify the benefits of medications in increasing survival and preventing recurrences.
